# Medical Mistrust and Adherence to Care Among a Heterogeneous Cohort of Women Living with HIV, Followed in a Large, U.S. Safety Net Clinic

**DOI:** 10.1089/heq.2020.0105

**Published:** 2021-09-24

**Authors:** Lunthita M. Duthely, Alex P. Sanchez-Covarrubias, Varsha Prabhakar, Megan R. Brown, Tanya E.S. Thomas, Emily K. Montgomerie, JoNell E. Potter

**Affiliations:** ^1^ Divisions of Research and Special Projects and Department of Obstetrics, Gynecology and Reproductive Sciences, University of Miami Miller School of Medicine, Miami, Florida, USA.; ^2^ Divisions of Gynecologic Oncology, Department of Obstetrics, Gynecology and Reproductive Sciences, University of Miami Miller School of Medicine, Miami, Florida, USA.; ^3^ Department of Medical Education, University of Miami Miller School of Medicine, Miami, Florida, USA.; ^4^ Division of Infectious Diseases, Department of Medicine, University of Miami Miller School of Medicine, Miami, Florida, USA.

**Keywords:** HIV/AIDS, clinics, adherence, medical mistrust, racial minority

## Abstract

**Purpose:** To explore the relationship between medical mistrust, as measured by the Group-Based Medical Mistrust (GBMM) scale, and HIV care adherence among a cohort of minority women receiving care in a U.S. safety net clinic.

**Methods:** English-, Spanish-, and Haitian Creole (Creole)-speaking patients with a recent history of nonadherence to care were surveyed.

**Results:** English speakers endorsed the highest level of mistrust, followed by Spanish speakers and Creole speakers. Creole speakers endorsed lower mistrust, lower suspicion of providers, and lower levels of “perceived health care disparities.” Higher mistrust was associated significantly with lower medication adherence, and lower rates of viral suppression (nonsignificant).

**Conclusion:** Understanding perceptions of medical care and the relationship to HIV care adherence is an important step to addressing negative health outcomes for ethnic minority women with HIV.

Clinical Trial Registration Number: NCT03738410.

## Introduction

Miami has the highest HIV transmission rate in the United States, yet only 61% of women living with HIV (WLWH) receive care.^1^ Residents are 90% ethnic/racial minorities, 58% foreign born, and 77% speak a primary language other than English.^2^ Medical mistrust, a factor related to nonadherence to HIV care,^3–9^ is a patient's belief that providers do not act in their best interest. Patients who trust their providers and medical systems, overall, have higher rates of appointment attendance, medication adherence, and physical well-being.^3–5^

Mistrust related to medical care has been characterized for different U.S. racial/ethnic groups. African Americans have experienced centuries of racial inferiority stereotypes and unethical experimentation (see Tuskegee Syphilis Experiments^10,11^; African American cadavers used for medical training^12^), which influenced the reason African Americans doubted information regarding the origins of HIV.^11^ Discrimination toward immigration status and language proficiency generated mistrust among U.S. Hispanics.^4^ Mistrust among Haitians may stem from discrimination, following the Centers for Disease Control's identification of Haitian ancestry as a risk factor for HIV acquisition early in the epidemic.^13^

Acculturation, a significant factor affecting health behavior and health literacy,^14,15^ is the process through which a person adopts new practices, beliefs, and identity from the dominant culture to which they have immigrated.^15,16^ Among many proxies for acculturation,^14^ preferred language^17,18^ is considered a robust surrogate for acculturation.^19^ Little is known about medical mistrust across multiple linguistic/cultural groups, who share the same health condition (e.g., HIV) and medical system (i.e., medical clinic). In the United States, a comparison of medical mistrust by language as an acculturation surrogate for WLWH has not yet been studied.

Considering Miami's ethnic/linguistic diversity, characterizing medical mistrust among subgroups may inform interventions to optimize HIV health care and outcomes. Preference for a language other than English may be indicative of limited English proficiency, which is associated with worse health outcomes for ethnic minority WLWH. Little is reported, however, on the linguistic minorities in the United States and their experiences of medical mistrust.^20–22^ Furthermore, only few studies in the existing medical mistrust literature reported on ethnic minority women or disaggregate findings by gender.^3–9^ Our primary objective was to measure medical mistrust variability among a clinic cohort of ethnic minority women, followed in a U.S. safety net clinic, with recent histories of nonadherence to HIV medical care. Considering the shared experiences of minority status and an HIV diagnosis that was managed in a public clinic, we hypothesized an equally high medical mistrust across the linguistic groups—English, Spanish, and Haitian Creole (Creole). Our secondary objective was to examine the association between mistrust and subjective and objective measures of nonadherence, among a convenient sample of ethnic minority WLWH followed in a Miami clinic. Given that more than one-third of the cohort was found to have a history of one or more mental health conditions, we also examined the association between mistrust and mental health history.

## Methods

### Participants

WLWH were surveyed as part of an ongoing study enrolling ethnic minority women with a recent history of nonadherence to care, defined as the following: HIV viral load (VL) >20 copies/mL, missed (without canceling/rescheduling) ≥1 of ≥2 scheduled visits (recent 12 months), or not taking antiretrovirals (ARV) as prescribed. Participants (*n*=54), recruited from an urban, HIV safety net clinic that serves 900 women annually, were surveyed in their preferred language (English, Spanish, Creole) and compensated for their time with grocery gift cards (see published protocol^23^). The clinic, situated in an academic medical center, provides gynecological, obstetrical, and primary care services to a predominantly low-income population of women receiving free or subsidized HIV medical and social support services. The primary author's Institutional Review Board (#20170287) approved data collection and results dissemination.

### Measures

#### Demographic Questionnaire

A study-created form captured self-reported age and race/ethnicity.

#### Group-Based Medical Mistrust Scale (self-report)

This scale measures minority group mistrust,^24^ provides an overall score (12–60), and includes three subscales: (1) suspicion of providers; (2) perceived health care disparities; and (3) perceived lack of support from health care providers. The Group-Based Medical Mistrust (GBMM), first validated with black and Latina women to assess barriers to breast cancer screening,^24^ has been used for HIV patients across both genders^3,5,6,9^ and among men^3,4,25,26^; one study, to-date,^8^ focused on WLWH. Individual GBMM subscales have been used as well, within the context of ARV medications among minorities with HIV.^27,28^

#### Three-Item Medication Adherence Scale (self-report; recent 30 days)

Subscales measure number of days taken (continuous, out of 30), frequency, and overall rating of adherence to ARV medications.^29^

#### Medical record data (recent 12 months)

HIV VL (copies/mL) was reduced to a categorical variable, representing suppression (<200 copies/mL). History of mental health conditions and illicit substance use was extracted from participants' medical records as well.

### Study exposures and outcomes

Primary outcomes were overall medical mistrust (median GBMM) and medical mistrust subscales of suspicion of providers, perceived health care disparities, and perceived lack of support from health care providers (GBMM subscales) by language. Total GBMM and GBMM subscale scores are summed on a five-point Likert scale, where higher scores indicated higher levels of medical mistrust. Secondary outcomes included the associations between GBMM and the three-item Medication Adherence Scale, VL suppression, and mental health history.

### Statistical analysis

Data were analyzed in SAS Studio 9.4 (SAS Institute Inc., Cary, NC), reporting frequencies, proportions, and chi-square *p*-values for categorical variables; means, standard deviations, medians, and interquartile ranges (IQRs) for continuous variables. As data were not uniformly distributed; the Kruskal–Wallis analysis (Kruskal–Wallis) tested overall GBMM and each subscale among language groups. The Wilcoxon–Mann–Whitney 2-sample rank sum (Wilcoxon–Mann–Whitney) tested associations between GBMM and (1) the three-item Medication Adherence Scale (Medication Adherence Scale); (2) VL; and (3) mental health condition. Due to data clustering toward higher values on the Medication Adherence Scale, two-level categorical variables were created for each subscale—specifically, days taken (30 vs. ≤29), frequency (“Always” vs. “Other”), and rating (“Excellent” vs. “Other”). Two-sided *p* values were reported, where *p*<0.05 was considered statistically significant.

## Results

Although 54 records were available, 3 records were excluded, due to incomplete key data—leaving a total of 51 complete records for analysis.

### Demographics and psychosocial profile

Participants, whose preferred languages were English (51%), Spanish (25%), and Creole (24%), were on average 45 years old. English speakers self-identified as follows: 89% black/African American and 11% Hispanic; one Spanish speaker self-identified as white, non-Hispanic; 100% of Creole speakers self-identified as black/African American. Thirty-seven percent of participants had history of a mental health condition. Significant differences were found when stratified by language group, where English speakers were the youngest and Spanish speakers were the oldest (*p*<0.002). A significantly higher proportion of English speakers were found to have a history of one or more mental health disorders, compared with Creole and Spanish speakers; Spanish speakers had the lowest proportion (*p*<0.05).

Other patterns emerged, where English speakers endorsed illicit drug use at higher proportions, compared with other linguistic groups; English speakers also endorsed lower medication adherence for two of the three adherence scales. Creole speakers had the lowest rate of documented viral suppression (67%), which was similar to English speakers (69%); Spanish speakers had the highest rate of viral suppression (92%) (Table 1).

**Table 1.** Participant Demographic and Psychosocial Profile

	Total N (%)	Creole 12 (24)	English 26 (51)	Spanish 13 (25)	p
Age (mean±SD)	44.6 (12.7)	48.92 (13.4)	38.23 (11.3)	53.31 (7.5)	0.0013
Race/ethnicity					—
White	1 (2)	—	—	1 (7)	
Black/African American	35 (69)	12 (100)	23 (89)	—	
Hispanic/Latinx	15 (29)	—	3 (11)	12 (93)	
Viral load (<200)	38 (75)	8 (67)	18 (69)	12 (92)	0.2359
Drug use	7 (14)	1 (8)	5 (19)	1 (8)	0.6527
Mental health history					0.0477
Major depressive/psychiatric disorder	19 (37)	3 (25)	14 (54)	2 (15)	
None noted	32 (63)	9 (75)	12 (46)	11 (85)	
Three-item medication adherence					
Days taken=30	31 (61)	8 (67)	15 (58)	8 (62)	0.8687
Frequency: “Always”	34 (67)	8 (67)	16 (62)	10 (77)	0.6814
Rating: “Excellent”	27 (53)	5 (42)	14 (54)	8 (62)	0.6616

SD, standard deviation.

### Medical mistrust

Participants overall had high mistrust compared with what has been reported for women living in rural regions of the United States (non-HIV^30^), yet similar to what was reported for urban U.S. women (non-HIV^10^). Specifically, median GBMM for the entire cohort was 29 (IQR 11)—21.5 for Creole speakers, 28 for Spanish speakers, and 32 for English speakers (Table 2). The differences across language groups were significant (Kruskal–Wallis, chi-square=10.7790, *p*=0.0046, df=2). *Post-hoc* analyses (Dwass-Steel-Critchlow-Fligners) showed Creole speakers endorsed significantly lower mistrust compared with English speakers (pairwise, two-sided; *p*=0.0067) and compared with Spanish speakers (pairwise, two-sided; *p*=0.0206) (Table 2). Stratification by GBMM subscales showed significant differences on the Suspicion subscale between Creole and English speakers (*p*=0.0473), with Creole speakers endorsing lower levels of suspicion (Table 2); and, for the Health Disparities subscale between Creole and Spanish speakers (*p*=0.0188), where Creole speakers perceived to a lesser degree disparities in how they were treated (Table 2).

**Table 2.** Median, Interquartile Range, and Kruskal–Wallis Results of GBMM Scores by Participant Language Group

GBMM subscales	Language	Median	IQR	n
Suspicion	Creole	6.00^*^, ^a^	5.50	12
	English	12.00^*^, ^a^	6.00	26
	Spanish	11.00^*^	6.00	13
Health care disparities	Creole	10.00^*^, ^b^	8.00	12
	English	11.00^*^	4.00	26
	Spanish	12.00^*^, ^b^	5.00	13
Lack of support	Creole	4.50	5.50	12
	English	8.00	3.00	26
	Spanish	7.00	3.00	13
Total GBMM	Creole	21.50^*^, ^c^,^d^	11.00	12
	English	32.00^*^,^c^	7.00	26
	Spanish	28.00 ^*^, ^d^	9.00	13

^*^ Kruskal–Wallis test comparing these groups demonstrated a statistically significant difference across medians (*p*<0.05).

a,b,c,d Same letter indicated significant difference across subgroups (*p*<0.05).

GBMM, Group-Based Medical Mistrust; IQR, interquartile range.

### Medical mistrust by ARV adherence, and VL suppression

Examined by ARV adherence, GBMM for the Medication Adherence Scale showed statistical significance between the frequency subscale (“Always” or “Other”) and median GBMM score for the entire cohort (28 vs. 34, *p*=0.0186). Within language groups, Creole speakers reporting “Always” taking ARV had a median GBMM score of 19.5, compared with those reporting taking ARV less often (“other”; median GBMM=26.5, *p*=0.0259). Associations between GBMM and other ARV adherence subscales (i.e., number of days, overall rating), as well as between GBMM and VL suppression were not significant (Table 3). In a sub-analysis, we then examined median GBMM across all languages, comparing Medication Adherence frequency (“Always” or “Other”) with viral suppression, and found no significant differences (*p* = 0.1197); however, those who endorsed “Always” taking their ARV, compared with not always, trended towards lower mistrust, regardless of viral suppression (Table 3).

**Table 3.** Median, Interquartile Range, and Kruskal–Wallis Results of Total GBMM Score, Across Linguistic Groups, by Medication Adherence, Viral Load Suppression, and Mental Health History

Variables	GBMM overall score: median (IQR)	GBMM Creole speakers: median (IQR)	GBMM English speakers: median (IQR)	GBMM Spanish speakers: median (IQR)
Three-item medication adherence				
Days taken				
30	28 (14)	19.5 (15)	31 (11)	27.5 (9.5)
≤29	31 (7)	24.5 (4.5)	34 (7)	32 (4)
Frequency				
Always	28 (11)^*^	19.5 (9.5)^**^	30 (7)	28 (9)
Other	34 (8)^*^	26.5 (16)^**^	35 (6)	32 (17)
Rating				
Excellent	28 (10)	19 (11)	31.5 (11)	27.5 (8)
Other	29.5 (8.5)	24 (8)	33 (6)	32 (7)
Viral load				
Not suppressed	32 (10)	24.5 (4.5)	34.5 (4)	37 (—)
Suppressed	28 (9)	19.5 (15)	30 (7)	28 (7.5)
Mental health condition (history)				
Yes	31 (9)	25 (8)	33 (7)	31.5 (11)
No	28 (10)	20 (12)	31.5 (6)	28 (9)

^*^ *p*<0.02; ^**^p<0.03.

### Medical mistrust by mental health history

A history of one or more mental health disorders (37% overall) correlated to higher GBMM scores (31 vs. 28). Stratified by language, the trend persisted; however, the Wilcoxon–Mann–Whitney test was not significant among languages (Table 3).

## Discussion

Among this cohort of English-, Spanish-, and Creole-speaking minority WLWH in Miami, medical mistrust was inversely and significantly related to adherence to ARV medications. Lower mistrust correlated with VL suppression among the three language groups (not significantly). Recent studies also reported that lower mistrust was associated with better ARV adherence^3,6^ and VL suppression.^8,9^ A median GBMM score of 29 mirrored what was reported for another cohort of urban women (non-HIV; GBMM=29),^10^ but higher than that reported for rural women (non-HIV; GBMM=19).^30^ Despite a history of nonadherence, the women in this cohort were still in HIV care, perhaps explaining why mistrust was not higher than hypothesized.

A linguistic group analysis showed that Creole speakers endorsed lower medical mistrust, compared with the other groups; Spanish speakers endorsed lower mistrust compared with English speakers, most of whom were African American. One study (men with HIV) reported lower mistrust for Hispanics compared with African Americans.^4^ In our study, Creole speakers were also less suspicious of medical providers and did not perceive disparities in health care as severely, compared with other groups. Interestingly, despite endorsing lower mistrust, Creole speakers were least likely to be virally suppressed (67%), which mirrored the English speakers (69%). For all linguistic groups, there was a trend of lower mistrust, overall, and for the individual mistrust subscales, which correlated to both better self-reported adherence and higher rates of viral suppression. Significant differences persisted, however, for Creole speakers only: creole speakers reporting “Always” taking ARV endorsed lower levels of medical mistrust.

Suspicion of medical providers and perceived differences in medical care were endorsed to a lesser degree by Creole speakers, compared with Spanish and English speakers—the majority of English speakers (89%) were of black race (non-Hispanic descent). No previously published U.S. studies reported on mistrust across the three linguistic groups. One study reported that Hispanics endorsed lower mistrust, compared with black non-Hispanics^4^; another reported that both black non-Hispanics and Hispanics endorsed higher mistrust, compared with white non-Hispanics.^30^

In Miami, Hispanics are a majority-minority and Spanish is the dominant spoken language. Assuming preferred language represented some measure of acculturation^14,15^ in our study, it is plausible that Creole speakers (who are a linguistic and ethnic minority group and have a shorter history of residency in Miami) were acculturated to a lesser degree, compared with the Spanish speakers, and may explain why, regarding mistrust, Spanish and English speakers are more similar to each other. Prior studies showed that mistrust correlated positively with acculturation among rural Latinos^31^; and white non-Hispanic women reported more dissatisfaction with health care, compared with black non-Hispanic women, which according to the authors, may have been influenced by socio-economic status.^30^ In our study, Spanish-speakers and English speakers differed with respect to viral suppression, where Spanish speakers who were significantly older and less likely to have a mental health disorder had the highest rate of viral suppression (92%). The limited size of the cohort did not permit higher order statistical tests to adjust for different factors (e.g., age, mental health status, language).

Regarding psychosocial history, nearly 40% of the cohort had a history of a mental health disorder, and as noted earlier, across all linguistic groups there was a nonsignificant trend toward higher mistrust among those with mental health disorder. In describing the three linguistic groups, significant differences were found, where English speakers had one or more mental health disorders and were the youngest; Spanish speakers had the lowest proportion of mental health disorders, were the oldest, and had the highest rate of viral suppression (92%). It is not uncommon that mental health conditions, substance use, and HIV co-occur.^3–5^ Although not significant, English speakers endorsed more illicit substance use, compared with the other linguistic groups; English speakers also endorsed lower medication adherence for two of the three adherence scales. Data gathered from a larger cohort would determine whether differences among the linguistic groups would be upheld, as well as clarify the relationships between these factors (age, mental health conditions, illicit drug use, medication adherence), medical mistrust, and viral suppression.

Given the limited cohort size, we recognize that findings are not generalizable to all ethnic minority WLWH in the United States. It was beyond the scope of this study to adjust for factors relevant to ethnic minorities that further measure acculturation (i.e., time in the United States if foreign born), and other factors related to adherence (i.e., length of time with HIV). A larger study sample would permit higher order comparisons; qualitative inquiries by linguistic and ethnic groups may elucidate observed differences. Despite these limitations, to our knowledge, this is the first study to report on medical mistrust and individual mistrust subscales (suspicion, perceived disparities in health care, lack of support) among WLWH, comparing multiple U.S.-based linguistic/ethnic minority groups.

Beyond measuring medical mistrust, health promoting interventions are needed to reduce the negative health outcomes associated with mistrust of individual providers and of medical systems^32^; patient-centered approaches may mitigate medical mistrust among ethnic minority patients in the United States; however, results have been mixed (see Ref.^33,34^). Patient navigators, used by large and underresourced clinics to link patients to services, have been implemented to address medical mistrust in different disease domains, including cancer and HIV, and results have been mixed. Navigators serve in multiple capacities, including linking patients to needed services and resources to manage their health conditions. In one U.S. study, patient navigators provided patient education to improve patient satisfaction, however, medical mistrust did not significantly decrease.^33^ Technology-based approaches, such as videos aimed at improving health literacy, have had mixed results, as well, in significantly reducing medical mistrust among ethnic minorities in the United States (see Ref.^34^). Nuanced approaches are needed to address medical mistrust among minority groups,^32^ in general, and specifically for persons living with HIV.^3,5,6,9^

## Conclusion

Among this cohort of ethnic minority WLWH at early stages of nonadherence to HIV care, higher medical mistrust was associated with lower ARV adherence. Differences by linguistic groups emerged for general medical mistrust, and within individual subscales. Creole speakers, who may have acculturated to a lesser degree, compared with Spanish speakers, due to Spanish being the dominantly spoken language in Miami, endorsed lower suspicion of medical providers and were less likely to endorse that they received medical care that was different, compared with what Spanish and English speakers endorsed. As the world aspires to end the HIV/AIDS epidemic, understanding perceptions of medical care and how these perceptions interfere with adherence to HIV care is an important consideration. Additional studies with larger cohorts, taking a mixed-methods approach, would expand on these findings for a better understanding of the relationship between medical mistrust, adherence to care, and viral suppression. Our findings highlight the need for more nuanced approaches to understanding adherence to HIV care, which go beyond an individual's ethnicity/race, and considerations of language—a potential proxy for acculturation. Our findings may also contribute to investigations that seek to mitigate the effects of medical mistrust on HIV health outcomes for ethnic minority subgroups.

## Abbreviations Used

ARV: antiretrovirals
GBMM: Group-Based Medical Mistrust
IQR: interquartile range
SD: standard deviation
VL: HIV viral load
WLWH: women living with HIV
